# Exosomal miR-146a-3p modulates neural stem cell fate through UCHL1 downregulation in spinal cord injury: implications for neuroregeneration: miR-146a-3p modulates NSC fate through UCHL1 downregulation

**DOI:** 10.3724/abbs.2025131

**Published:** 2025-09-15

**Authors:** Ying Gao, Ziwen Shi, Suhe Zhang, Zhiqiang Wu, Tao Zhou, Pinghui Zhou, Weiwei Chu, Xuyi Wang

**Affiliations:** 1 Department of Anesthesiology the First Affiliated Hospital of Bengbu Medical University Bengbu 233004 China; 2 Department of Orthopaedics the First Affiliated Hospital of Bengbu Medical University Bengbu 233004 China; 3 Anhui Province Key Laboratory of Tissue Transplantation Bengbu Medical University Bengbu 233000 China; 4 Department of Plastic Surgery the First Affiliated Hospital of Bengbu Medical University Bengbu 233004 China

**Keywords:** spinal cord injury, cerebrospinal fluid, exosome, miR-146a-3p, UCHL1, neural stem cell

## Abstract

Spinal cord injury (SCI) poses a substantial challenge within the field of regenerative medicine, primarily because of its high incidence of disability and the paucity of effective therapeutic interventions. This study explores the involvement of cerebrospinal fluid (CSF) exosomes in the differentiation of neural stem cells (NSCs) following SCI, with a particular emphasis on the miR-146a-3p/UCHL1 signaling axis. Using an SCI animal model, we analyze the molecular composition of CSF and its influence on NSC fate through bioinformatics approaches, dual-luciferase reporter assays, and *in vitro* differentiation experiments. Differential expression analyses reveal a significant upregulation of miR-146a-3p in CSF-derived exosomes post-SCI, which directly targets and suppresses UCHL1, a pivotal regulator of neuronal differentiation. Overexpression of UCHL1 facilitates the differentiation of NSCs into neurons and enhances functional recovery, whereas its downregulation leads to increased astrocytic differentiation and fibrotic scar formation. These findings are corroborated through immunofluorescence, western blot analysis, and behavioral assessments. In summary, our study identifies miR-146a-3p as a critical regulator in SCI, offering novel insights into the role of microRNAs in modulating neural stem cell fate and promoting neuronal regeneration. Our study highlights the pivotal role of CSF exosomal miRNAs in determining NSC fate, paving the way for systemic interventions in injured spinal cord injury repair. These findings underscore the importance of exosomal miRNAs in modulating the spinal cord microenvironment, potentially leading to novel strategies for enhancing functional recovery in SCI patients.

## Introduction

Spinal cord injury (SCI) represents a severe disorder of the central nervous system (CNS) that is characterized by significant mortality and disability rates, thereby exerting considerable burdens on patients, their families, and healthcare systems globally. Globally, the annual incidence of SCI is estimated to be approximately 13 cases per 100,000 individuals. A published study reported that there were approximately 900,000 new cases of SCI worldwide in 2016. The incidence rates of SCI vary considerably across different countries and regions and are attributable to factors such as traffic accidents, violent incidents, and occupational safety [1]. In mainland China, more than one million individuals are affected by SCI, with approximately 120,000 new cases reported annually [2]. An epidemiological survey revealed that SCI predominantly impacts manual laborers, with a rising incidence observed among students and educators [3]. Notably, the primary etiology of SCI is often attributed to its occurrence alongside injuries to other major organs, including traumatic brain injury (TBI) and trauma to the chest, abdomen, and musculoskeletal system. This polytraumatic context can substantially impact the recovery trajectories of individuals with SCI [4]. Furthermore, the predominant causes of SCI include motor vehicle accidents, falls, sports-related unintentional injuries, and occupational hazards. For example, a study revealed that children engaging in backbends during dance activities represented a significant cause of SCI [5]. Rapid urbanization and an increased prevalence of automobiles are anticipated to increase the incidence of SCI, thereby posing a significant public health challenge. The high rates of disability associated with SCI impose substantial financial burdens and cause considerable suffering for both patients and their families [6]. The management of SCI continues to be a complex and challenging field. Despite extensive ongoing research, effective treatments that can fully restore function have yet to be discovered [7].

Despite the existence of abundant neural stem cells (NSCs) within the central canal of the spinal cord, the microenvironment following injury is not conducive to neuronal differentiation. Astrocytes form a glial scar at the site of injury, which plays a critical role in the healing process after SCI. However, this glial scar can also inhibit axonal regeneration [8]. Cerebrospinal fluid (CSF), which surrounds the entire brain and spinal cord, is essential for maintaining CNS homeostasis [9]. Injury to the CNS modifies the composition and concentration of active substances within the CSF, thereby directly affecting NSC activation and differentiation [10]. For example, elevated levels of brain-derived neurotrophic factor (BDNF) in CSF following TBI have been associated with increased proliferation of NSCs and their differentiation into neuroblastocytes [11]. Our previous studies demonstrated that normal CSF facilitates the differentiation of NSCs toward neuronal lineages, whereas CSF derived from injured spinal cords promotes glial differentiation [12]. Nevertheless, the precise molecular targets and mechanisms by which injured CSF influences NSC fate decisions remain inadequately understood.

Quiescent NSCs are characterized by low expressions of genes related to the ubiquitin-proteasome system. Dysregulation of intracellular proteins can result in the accumulation of protein aggregates, thereby impairing NSC activation [13]. Ubiquitin carboxyl-terminal hydrolase L1 (UCHL1), which is highly expressed in the nervous system, plays a crucial role in ubiquitin recycling and has been implicated in several neurological disorders [ 14 , 15].

CSF is enriched with exosomes derived primarily from neurons, astrocytes, oligodendrocytes, and microglia [16]. These exosomes play pivotal roles in intercellular communication within the nervous system and are integral to various neural repair mechanisms. These functions include the promotion of neuronal regeneration, the modulation of inflammatory responses, the facilitation of angiogenesis, the regulation of cellular signaling pathways, drug delivery, and the support of neurite remodeling, among other processes [17]. The microRNA (miRNA) profiles within CSF exhibit considerable variability across CNS injuries and various diseases, highlighting their stability and diagnostic potential. Following nerve injury, several miRNAs, including miR-9-5p, miR-128-3p, miR-9-3p, miR-124-3p, miR-204-5p, and miR-338-3p, are notably elevated in the CSF [ 18, 19]. A seminal study published in Nature elucidated the regulation of NSCs by miRNAs encapsulated within CSF exosomes. Through the use of Cy3-labelled miRNAs co-cultured with NSCs, the study confirmed that exosomal miRNAs can directly target NSCs. Furthermore, the introduction of exosomes derived from young mice into the CSF has been demonstrated to reduce the expression of inflammatory factors and promote the rejuvenation and functionality of NSC populations [20]. In addition, CSF miRNAs, including miR-125b [21], miR-222 [22], and let-7b [23], have been implicated in neural development, neurodegenerative diseases, and nerve injury repair by modulating gene expression in neural stem cells and influencing their biological behaviors, such as proliferation, differentiation, and migration [24].

To further investigate the underlying mechanisms of UCHL1 downregulation following SCI, we investigated the expression profiles of miRNAs within CSF exosomes that potentially target UCHL1. Our screening revealed that miR-146a-3p was significantly upregulated following SCI and was capable of directly inhibiting UCHL1 expression. In response to the urgent demand for innovative therapeutic strategies for SCI, which affects over one million individuals in China alone, our research departs from conventional methodologies that concentrate solely on the injury site. Instead, we focus on the CSF, a critical component influencing the CNS microenvironment. Our findings revealed that injured CSF markedly suppressed UCHL1 expression, thereby obstructing the differentiation of NSCs into neurons and compromising neural regeneration. Our study identified miR-146a-3p in CSF exosomes as a critical regulator that targets and inhibits UCHL1 following SCI, thereby elucidating a novel molecular mechanism contributing to the challenges associated with neuronal regeneration post-injury. We propose to further investigate the origin of miR-146a-3p and delineate the downstream molecular pathways associated with the miR-146a-3p/UCHL1 axis. Understanding these mechanisms through the lens of CSF-mediated microenvironmental changes holds substantial promise for the development of therapeutic interventions aimed at enhancing neural regeneration.

## Materials and Methods

### Animal models and experimental groups

Adult male Sprague-Dawley (SD) rats, aged 8–10 weeks and weighing 250–300 g, were procured from the Anhui Experimental Animal Center (Hefei, China). The animals were maintained under standard laboratory conditions, which included a 12/12-h light/dark cycle and ad libitum access to food and water. The animal program received approval from the Animal Ethics Committee of Bengbu Medical University in Anhui Province, China (IACUC-2305016).

The rats were randomly allocated to the following experimental groups: (1) sham-operated group: this group underwent laminectomy without sustaining SCI; and (2) SCI group: this group underwent complete transection at the T10 spinal cord level. The concentrations of various reagents used in this study were primarily determined on the basis of the manufacturer’s instructions. The experimental groups were administered with lentivirus (LV) vectors to overexpress UCHL1 following SCI (UCHL1-LV) or null control LV vectors (Null-LV). Additionally, the SCI group received treatment with UCHL1 recombinant protein (ab269110; Abcam, Cambridge, UK), following the protocol outlined in the product manual and our previous study [12], which involved administering with 10 μg in 25 μL of growth factor-reduced Matrigel (CLS356231; Merck, Darmstadt, Germany), hereafter referred to as UCHL1 RP. Another subgroup received a 24-h treatment with a 5-μM UCHL1 inhibitor, LDN57444 (HY-18637; MedChemExpress, Monmouth Junction, USA), and 25 μL of growth factor-reduced Matrigel following SCI. The inhibitor was dissolved in dimethyl sulfoxide (DMSO) to achieve the desired concentration and was incorporated into the culture medium. Furthermore, a subgroup was treated with either a miR-146a-3p mimic (5 nmol mimic mixed with liposomes dissolved in 25 μL of Matrigel) or a miR-146a-3p inhibitor (5 nmol inhibitor mixed with liposomes dissolved in 25 μL of Matrigel) post-SCI. The lentiviral vectors were procured from Cyagen Biosciences (Suzhou, China), while the miR-146a-3p mimic (5′-CGAGAACTGAATTCCATGGGTT-3′) and inhibitor (5′-AACCCATGGATTCAGTTCTC-3′) were obtained from Genechem (Shanghai, China).

### Induction of SCI and collection of CSFs

The rats were anaesthetized via the intraperitoneal injection of 3% pentobarbital sodium at a dosage of 30 mg/kg. Laminectomy at the T10 vertebral level was performed to expose the spinal cord. To induce SCI, complete transection was performed at the T10 vertebral level to ensure thoroughness of the injury. In contrast, sham-operated rats underwent laminectomy without transection of the spinal cord. At 24 h post-injury, CSF was collected via cisterna magna puncture. SD rats were anaesthetized, and their heads were secured in a stereotaxic frame. A midline incision was made between the ears to expose the deep muscles, which were retracted to clear the fascia. The cisterna magna, located at the lower margin of the occipital bone, was identified. A capillary glass pipette was carefully inserted into this region. Upon encountering a loss of resistance, indicating entry into the cisterna magna, the CSF was gently aspirated.

### Isolation, culture, and differentiation of NSCs

Endogenous NSCs were extracted from the subventricular zones of adult rats following an established protocol [12]. The cells were cultured in proliferation medium composed of DMEM/F12 supplemented with 2% B27 (17504044; Thermo Fisher Scientific, Waltham, USA), 20 ng/mL EGF (AF-100-15; Gibco, Waltham, USA), and 20 ng/mL bFGF (100-18B; Gibco). For the differentiation assays, NSCs were seeded onto dishes coated with poly-L-lysine (0.1 mg/mL; P2636; Sigma-Aldrich, St Louis, USA) and cultured in differentiation medium composed of DMEM/F12 (11330032; Gibco) supplemented with 1% fetal bovine serum. This medium was further enriched with 10% normal CSF or CSF derived from acute SCI and collected 24 h post-injury. After a seven-day incubation period, the cells were fixed and subjected to immunofluorescence staining to detect tubulin-β3, a neuronal marker, and glial fibrillary acidic protein (GFAP), an astrocyte marker. Furthermore, EdU staining was performed following the manufacturer’s protocol to label proliferating cells. The cells were incubated with EdU (10 μM) for 2 h, followed by fixation and detection using the Click-iT EdU Alexa Fluor 594 Imaging Kit (C10337; Invitrogen, Waltham, USA).

### Exosome isolation

Exosomes were extracted from both NSC-conditioned media and CSF samples through differential ultracentrifugation. The samples were subjected to sequential centrifugation at 300 *g* for 10 min, followed by centrifugation at 2000 g for 20 min and 10,000 *g* for 30 min to remove cells and debris. The resulting supernatants were ultracentrifuged at 100,000 *g* for 70 min. The exosome pellets were subsequently washed with phosphate-buffered saline (PBS) and subjected to additional centrifugation at 100,000 *g* for 70 min.

### Transmission electron microscopy assay

Exosomes were gently vortexed and fixed with 2% formaldehyde for 5 min. Next, the glow-discharged formvar/carbon-coated 200-mesh grids were prepared for 1 min to improve their hydrophilicity. A volume of 5 μL of the sample was placed onto each grid, allowed to incubate for 1 min, and any excess liquid was carefully removed using filter paper. For negative staining, the grids were covered with 1% uranyl acetate (or 3% phosphotungstic acid), briefly rinsed with ultrapure water, and left to air-dry for 10 min. Finally, the grids were inserted into a transmission electron microscopy (TEM) operating at 100 kV, and high-magnification images were captured to observe cup-shaped vesicles ranging from 30 to 200 nm in diameter.

### Nanoparticle tracking analysis

Exosomes were centrifuged at 10,000 *g* for 5 min to ensure uniformity. The sample was then diluted 100 times in 0.22 μm-filtered 1× PBS to reach a final concentration of 10 ^6^ particles/mL. Before measuring the sample, the NanoSight chamber was rinsed first with 1 mL of deionized water and then with 1 mL of PBS. After calibration, 300–400 μL of the diluted exosome suspension was introduced into the chamber at 25°C, using a 488 nm laser and a viscosity setting corresponding to PBS (0.887 cP). Three 60-s videos were recorded for each sample, ensuring that each video frame captured at least 20 particle tracks for reliable statistical analysis. Data were analyzed using ZetaView software with the following settings: a detection threshold of 5–7, a minimum track length of 5, and a bin size ranging from 1 to 10 nm. The resulting size distribution, mean diameter, and concentration (particles/mL) were exported as CSV files for further visualization in GraphPad.

### Western blot analysis

Proteins were extracted from cells or tissues using RIPA buffer (P0013C; Beyotime, Shanghai, China). Protein concentration was measured with the BCA Protein Assay Kit (P0012S; Beyotime). Samples were then denatured in SDS buffer at 95°C for a few minutes. The proteins were separated by SDS-PAGE and transferred onto methanol-activated PVDF membranes (Millipore, Mississauga, Canada) at 100 V for 1 h. The membranes were blocked with 5% non-fat milk in TBST for 1 h at room temperature, followed by an overnight incubation at 4°C with gentle shaking using primary antibodies against UCHL1 (13179; Cell Signaling Technology, Boston, USA), Tubulin-β3 (ab78078; Abcam), GFAP (3670; Cell Signaling Technology), CNPase (2986; Cell Signaling Technology), GAPDH (39-8600; Invitrogen), NF-200 (#30564; Cell Signaling Technology), Nestin (ab105389; Abcam), β-actin (ab8226; Abcam), CD63 (PA5-92370; Invitrogen), and CD81 (MA5-3233; Invitrogen). After incubation with primary antibodies, the membranes were washed thoroughly and then incubated with the corresponding HRP-conjugated secondary antibodies (P0948, A0216; Beyotime) at room temperature. Finally, immunoreactive bands were detected using an ECL substrate, and images were captured using an appropriate imaging system.

### miRNA extraction and quantitative PCR

Total RNA, including miRNAs, was isolated from the exosomes using the miRNeasy Mini Kit (217084; Qiagen, Venlo, Netherlands). The miRNA was reverse transcribed with specific stem-loop primers, and quantitative PCR (qPCR) was performed with SYBR qPCR Master Mix (Q712-02; Vazyme, Nanjing, China). The levels of miRNAs were normalized to those of *U6* small nuclear RNA via the 2 ^–ΔΔCt^ method. The primer sequences for the rat genes were as follows: *UCHL1* (F: 5′-AACCCCGAGATGCTGAAC-3′; R: 5′-GGCTGCCTGAATGGCCTC-3′), *α-tubulin* (F: 5′-CTCGCATCCACTTCCCTC-3′; R: 5′-ATGCCCTCACCCACGTAC-3′), *let-7d-5p* (F: 5′-AGAGGTAGTAGGTTGCATAG-3′; R: 5′-GAACATGTCTGCGTATCTC-3′), *miR-146a-3p* (F: 5′-ACCUGUGAAGUUCAGUUCUUU-3′; R: 5′-CAGTGCCTGT CGTGGAGT-3′), *miR-204-3p* (F: 5′-GTCGTGAGACTGGCCAGCAGG-3′; R: 5’-GACATGGAGTGAGTGACAGG-3′), *miR-320-3p* (F: 5′-ACACTCCAGCTGGGAAAAGCTGGGTTGAGA-3′; R: 5′-TGGTGTCGTGGAGTCG-3′), and *U6* (F: 5′-CTCGCTTCGGCAGCACA-3′; R: 5′-AACGCTTCACGAATTTGCGT-3′).

### Dual-luciferase reporter assay

The 3′ untranslated region (3′UTR) of *UCHL1* mRNA, which includes the wild type (WT) or Mut predicted binding sites for miR-146a-3p, was cloned and inserted into the pmirGLO vector (Promega, Madison, USA). HEK293T cells were co-transfected with this reporter construct and either a miR-146a-3p mimic or a negative control using Lipofectamine 2000 (Invitrogen). After 48 h, luciferase activity was quantified using the Dual-Luciferase Reporter Assay System (Promega) in accordance with the manufacturer’s protocol.

### *In vitro* modulation of UCHL1 and miR-146a-3p expression

NSCs were transduced with lentiviral vectors to overexpress either UCHL1 or the null vector provided by Cyagen Biosciences. For the modulation of miR-146a-3p, NSCs were transfected with miR-146a-3p mimics, inhibitors, or their respective controls via Lipofectamine RNAiMAX (Invitrogen). Stable cell lines were subsequently selected using puromycin at a concentration of 2 μg/mL. The efficiency of differentiation into neurons and astrocytes was evaluated post-induction through immunofluorescence staining and western blot analysis.

### Behavioral assessments

Motor function was systematically evaluated via the Basso, Beattie, and Bresnahan (BBB) locomotor rating scale on days 1, 7, 14, 21, and 28 postinjury. The BBB locomotor rating scale is a widely used system to assess motor function recovery after SCI in rats. The scale evaluates hindlimb movement, coordination, and posture. It ranges from 0 (no movement) to 21 (normal movement), with specific criteria for each score. Footprint analysis and inclined grid tests were conducted to evaluate functional axonal conduction and motor coordination. Footprints and foot angles were recorded using needle electrodes inserted into the hindlimb muscles, with stimulation administered to the motor cortex.

### Immunohistochemical analysis

At specified intervals, the rats were perfused transcardially with cold PBS and fixed with 4% paraformaldehyde. Spinal cord tissues were then collected, post-fixed, cryoprotected in 30% sucrose, and sectioned at a thickness of 10 μm. The sections were subjected to immunofluorescence staining with primary antibodies against UCHL1 (1:250; ab108986; Abcam), GFAP (1:250; 3670; Cell Signaling Technology), neurofilament 200 (NF200; 1:250; 13-1300; Thermo Fisher Scientific), tubulin-β3 (1:250; ab78078; Abcam), and CNPase (1:250; sc-166558; Santa Cruz Biotechnology, Santa Cruz, USA). After being washed in PBS, the cells and sections were incubated with Alexa Fluor-conjugated secondary antibodies (Alexa Fluor 488: 1:1000; A-11094; Invitrogen; and Alexa Fluor 594: 1:1000; A-11037; Invitrogen) and counterstained with DAPI (C1102; Beyotime). Fluorescence images were captured via a Zeiss LSM 880 confocal laser scanning microscope (Zeiss, Wetzlar, Germany), followed by quantitative analysis via ImageJ software.

### Hematoxylin and eosin staining

Spinal cord samples were fixed, dehydrated through a graded series of alcohol solutions, cleared in xylene, infiltrated with molten paraffin wax, embedded, cooled, sectioned at 10-μm thickness, floated on warm water, mounted onto glass slides, and subsequently dried. Paraffin sections of the spinal cord were dewaxed in xylene and rehydrated through a series of graded alcohol solutions to water. The nuclei were stained with hematoxylin, followed by rinsing and differentiation in acid-alcohol. The sections were then blued in an alkaline solution and rinsed again. The cytoplasm was counterstained with eosin, after which the sections were briefly rinsed. Subsequently, the tissue sections were dehydrated using ascending concentrations of alcohol, cleared in xylene, and mounted with a coverslip and mounting medium.

### Bioinformatics analysis

Raw sequencing reads from the public dataset GSE19890 (GEO) were downloaded and quality-filtered with FastQC and Trimmomatic. Differentially expressed miRNAs (|log2 FC| ≥ 1, FDR < 0.05) were identified by DESeq2. Putative target genes were predicted with miRWalk 3.0 ( http://mirwalk.umm.uni-heidelberg.de), intersecting results from TargetScan, miRDB and RNA22; only targets predicted by ≥ 3 algorithms were retained for downstream GO and KEGG enrichment analyses in clusterProfiler.

### Statistical analysis

The data are expressed as the mean ± standard error of the mean (SEM). Statistical analyses were performed via GraphPad Prism version 8.0. Comparisons between two groups were conducted via the unpaired Student’s *t* test, whereas comparisons among multiple groups were executed via one-way or two-way analysis of variance (ANOVA), followed by Tukey’s post hoc test. A *P* value of less than 0.05 was considered indicative of statistical significance.

## Results

### Downregulation of UCHL1 expression following spinal cord injury

**Figure 1:**
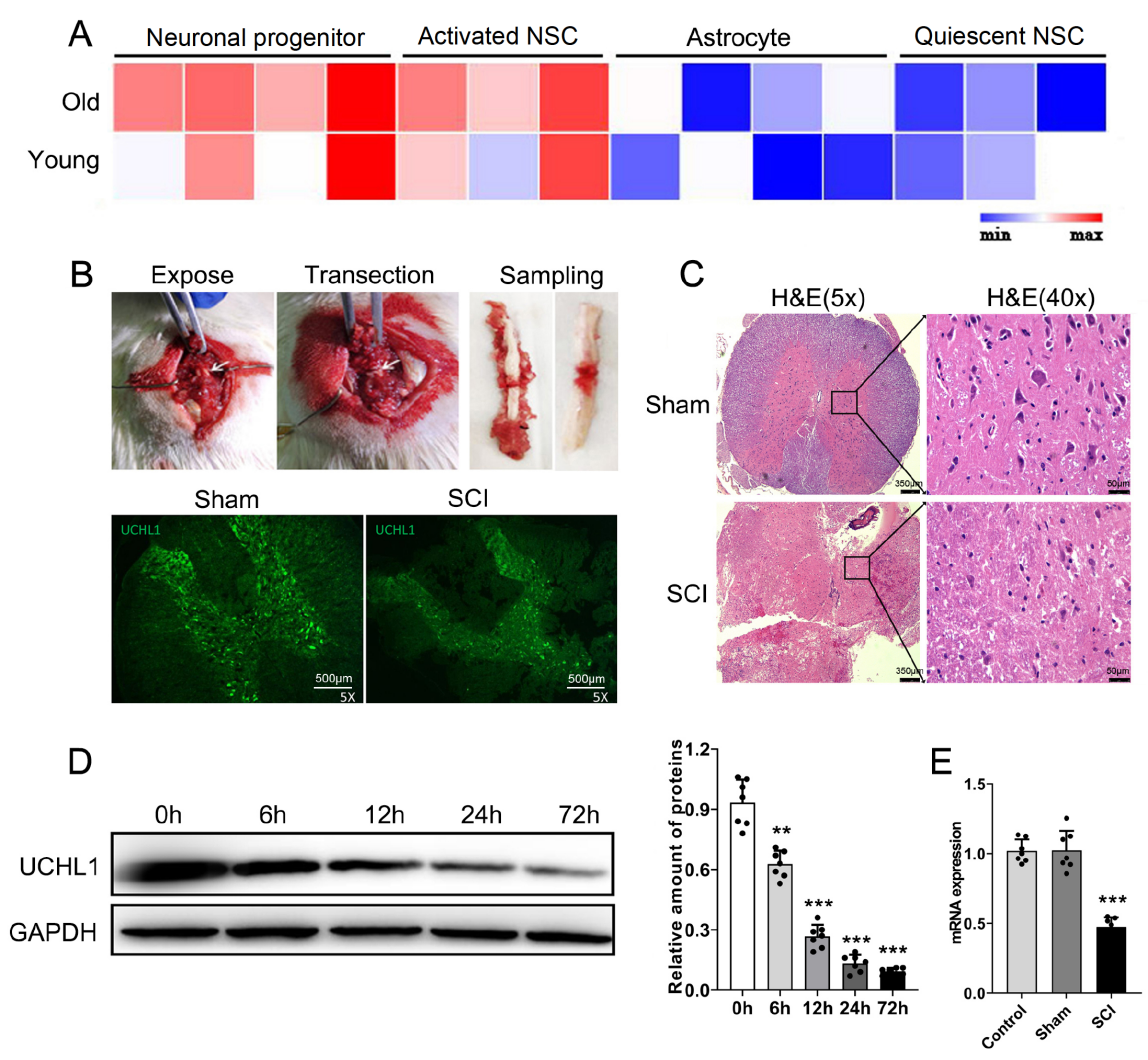
Figure 1 Expression profile of UCHL1 post-SCI (A) UCHL1 expression is markedly elevated in neuronal progenitor cells and activated NSCs compared with astrocytes and quiescent NSCs. (B) A rat model with complete spinal cord transection was constructed, and immunofluorescence staining for UCHL1 was performed 24 h post-surgery. Scale bar = 500 μm. (C) H&E staining provides detailed insights into spinal cord integrity and neural density. 5×: scale bar = 350 μm; 40×: scale bar = 50 μm. (D) Western blot analysis and (E) qRT-PCR confirmed the downregulation of UCHL1 expression following SCI. ** P < 0.01, ***P < 0.001 versus 0 h. n = 7 per group. UCHL1 mRNA expression was detected 72 h post-surgery. *P < 0.05 versus the control. n = 7 per group.

To identify a more effective approach for activating NSCs, we analyzed the transcriptomic differences between quiescent and activated NSCs via data from the ArrayExpress database (accession number: E-MTAB-5172 [13]). The results demonstrated that UCHL1 levels in activated NSCs are significantly elevated compared with those in quiescent NSCs, regardless of whether the cells are derived from young or aged samples ( Figure 1A). In the experimental procedure, complete spinal cord transection was conducted on the rats. Immunofluorescence analysis performed 24 h posttransection revealed a marked reduction in UCHL1 expression levels ( Figure 1B). H&E staining revealed compromised spinal cord integrity and a substantial decrease in neuronal density ( Figure 1C). Following surgical intervention, UCHL1 protein levels progressively decreased, reaching only 18.5% of baseline levels by 72 h post-operation ( Figure 1D). Concurrently, the mRNA expression levels were reduced to 47.6% of the baseline values ( Figure 1E). Our study suggested that SCI leads to a marked reduction in UCHL1 levels, which may hinder neuronal regeneration. However, further studies are needed to establish the precise mechanism involved.

### UCHL1 facilitates NSC differentiation into neurons

**Figure 2:**
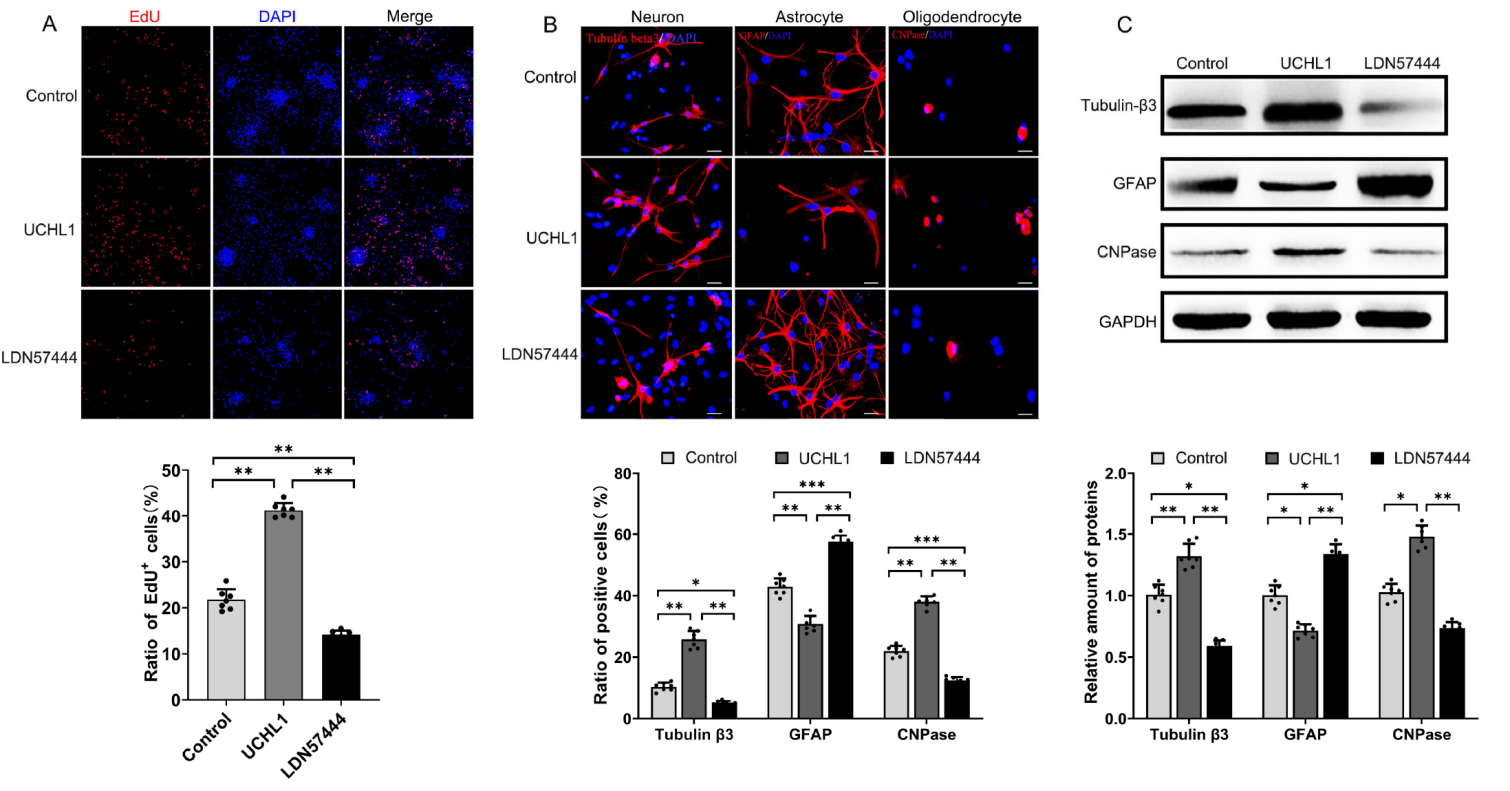
Figure 2 UCHL1 promotes NSC differentiation into neurons (A) NSCs were transfected with control lentivirus or UCHL1-overexpressing lentivirus or treated with a UCHL1 inhibitor (LDN57444, 5 μM) for 7 days. An EdU assay was used to evaluate their proliferative activity. (B,C) NSCs were cultured in medium containing 1% FBS to induce differentiation for 14 days. Immunofluorescence staining (B) and western blot analysis (C) were conducted with antibodies against Tubulin-β3, GFAP, and CNPase to evaluate the extent of NSC differentiation into their respective progeny cell types. Scale bar = 20 μm. * P < 0.05, **P < 0.01, ***P < 0.001. n = 7 per group.

In our previous study, we successfully engineered lentiviral vectors to facilitate the overexpression of UCHL1 [12]. These lentiviruses designed for UCHL1 overexpression were used to transfect NSCs *in vitro*. EdU staining was used to measure the proliferation of NSCs. These results indicated that UCHL1 overexpression significantly enhances the proliferation rates of NSCs. LDN57444 treatment reduced the proliferation rate, as indicated by the lower percentage of EdU ^+^ cells than in the UCHL1-overexpressing group. These findings suggested that inhibition of UCHL1 may impede the proliferation of NSCs and their ability to differentiate into neurons, astrocytes, and oligodendrocytes ( Figure 2A). Additionally, immunofluorescence assays revealed the differentiation of NSCs into different lineages (neurons, astrocytes, and oligodendrocytes). These findings demonstrated that UCHL1 promotes NSC differentiation toward neurons and oligodendrocytes while concurrently inhibiting astrocytic differentiation. Compared with UCHL1 overexpression, LDN57444 treatment led to a decrease in the differentiation of these lineages, particularly neurons. Tubulin β3-positive cells (neurons) and GFAP-positive cells (astrocytes) were significantly reduced, indicating that LDN57444 affects the differentiation potential of NSCs. The expression of the oligodendrocyte differentiation marker CNPase was also lower in the LDN57444 treatment group than in the UCHL1 overexpression group, suggesting that LDN57444 reduces the potential of NSCs to differentiate into oligodendrocytes ( Figure 2B). Western blot analysis of marker protein for neurons (Tubulin-β3), astrocytes (GFAP), and oligodendrocytes (CNPase) further confirmed that UCHL1 promotes neuronal and oligodendrocyte differentiation while inhibiting astrocyte differentiation. Compared with UCHL1 overexpression, LDN57444 treatment reduced the expression levels of Tubulin-β3 and CNPase ( Figure 2C). Collectively, these data suggest the potential role of UCHL1 in promoting neural regeneration, while LDN57444 inhibits NSC differentiation and may also modulate the function of neural progenitor cells by blocking specific signaling pathways.

### Overexpression of UCHL1 enhances neuronal regeneration post-SCI

**Figure 3:**
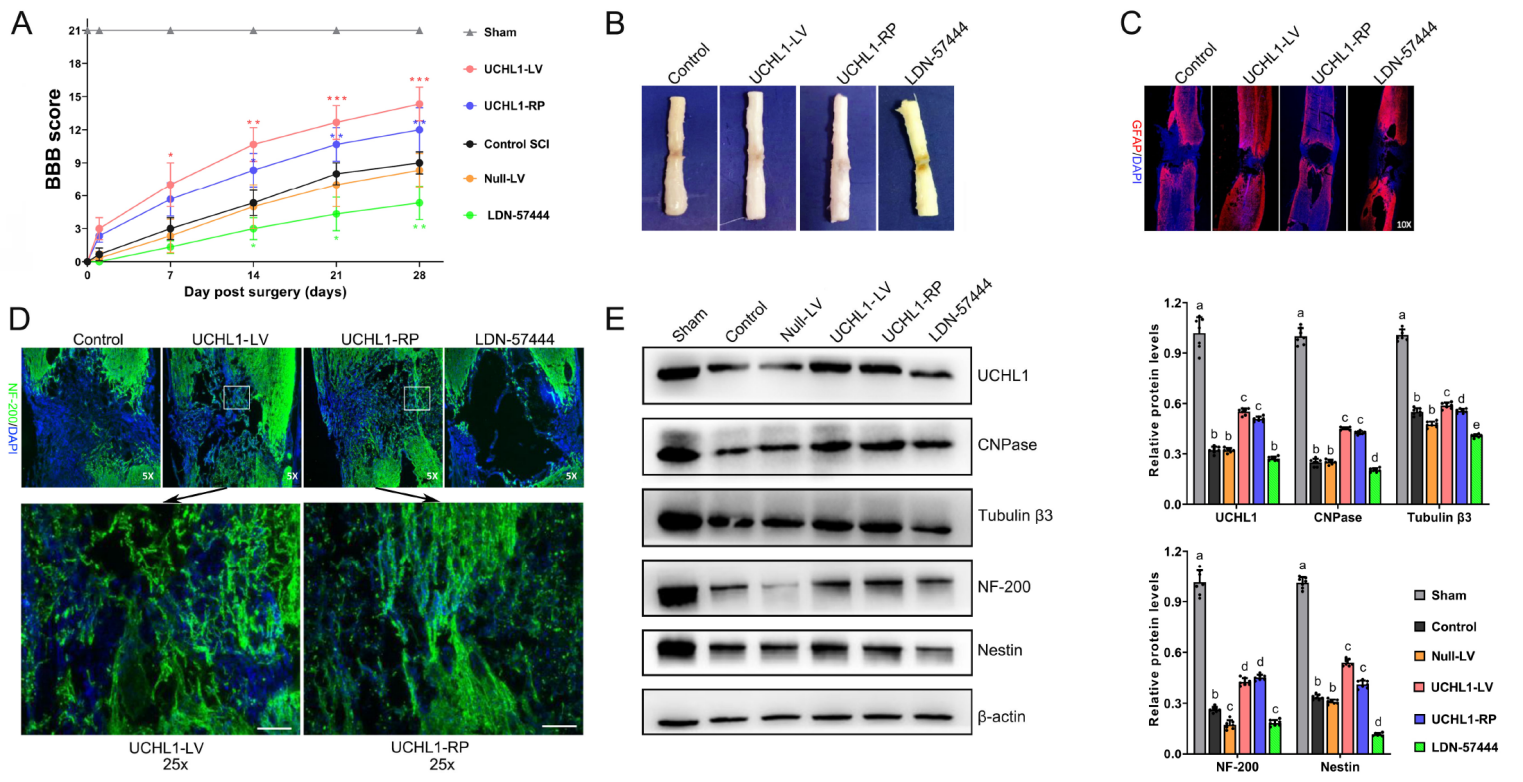
Figure 3 UCHL1 enhances neuronal regeneration post-SCI (A) Motor function assessments were conducted 21 days after treatment via the BBB scoring system, which revealed significantly enhanced recovery in the groups treated with UCHL1 overexpression and recombinant protein compared with the control groups. * P < 0.05, **P < 0.01, ***P < 0.001 versus the control-SCI group. (B) Morphological analysis of the spinal cord tissue. (C,D) Immunofluorescence staining for GFAP and NF-200 confirmed enhanced neurotrauma repair and neuronal regeneration at the injury site. Scale bar = 100 μm. (E) Western blot analysis of UCHL1, CNPase (oligodendrocyte marker), Tubulin-β3 (mature neuron marker), NF-200 (de novo neuron marker), and Nestin (NSC marker) expresssions. Different groups of lowercase letters (a, b, c, d, e) represent significant differences ( P < 0.05). n = 6 per group.

Twelve hours following spinal cord transection, the rats were treated with UCHL1-overexpressing lentivirus, recombinant protein, or the UCHL1 inhibitor LDN57444. The BBB score results demonstrated that UCHL1 overexpression and recombinant protein substantially enhanced motor function recovery, whereas the UCHL1 inhibitor LDN57444 suppressed motor function ( Figure 3A). Further confirmation of axon regeneration using additional markers, such as GFAP for astrocyte activation, is ongoing. Morphological analysis of the spinal cord samples collected at 21 days revealed that spinal cord recovery improved in both groups. However, the spinal cord lesions in the inhibitor group were notably larger than those in the control group ( Figure 3B,C).

The *de novo* neurons (NF-200 positive) were labelled using immunofluorescence staining. The results demonstrated that both the UCHL1 lentivirus and the recombinant proteome promoted significant nerve regeneration at the injury site, with some *de novo* neurons reestablishing connections between the rostral and caudal segments of the transected spinal cord. In contrast, the LDN57444 group exhibited the poorest nerve regeneration, with substantial neural deficits persisting at the injury site ( Figure 3D). Western blot analysis revealed consistent findings, demonstrating that UCHL1 overexpression and recombinant protein significantly increased the expression levels of UCHL1, the oligodendrocyte marker CNPase, the mature neuron marker Tubulin-β3, the *de novo* neuron marker NF-200, and the NSC marker Nestin in the spinal cords of SCI rats ( Figure 3E). These changes collectively contribute to neuronal regeneration and functional recovery following SCI.

### SCI CSF inhibits NSC differentiation into neurons

**Figure 4:**
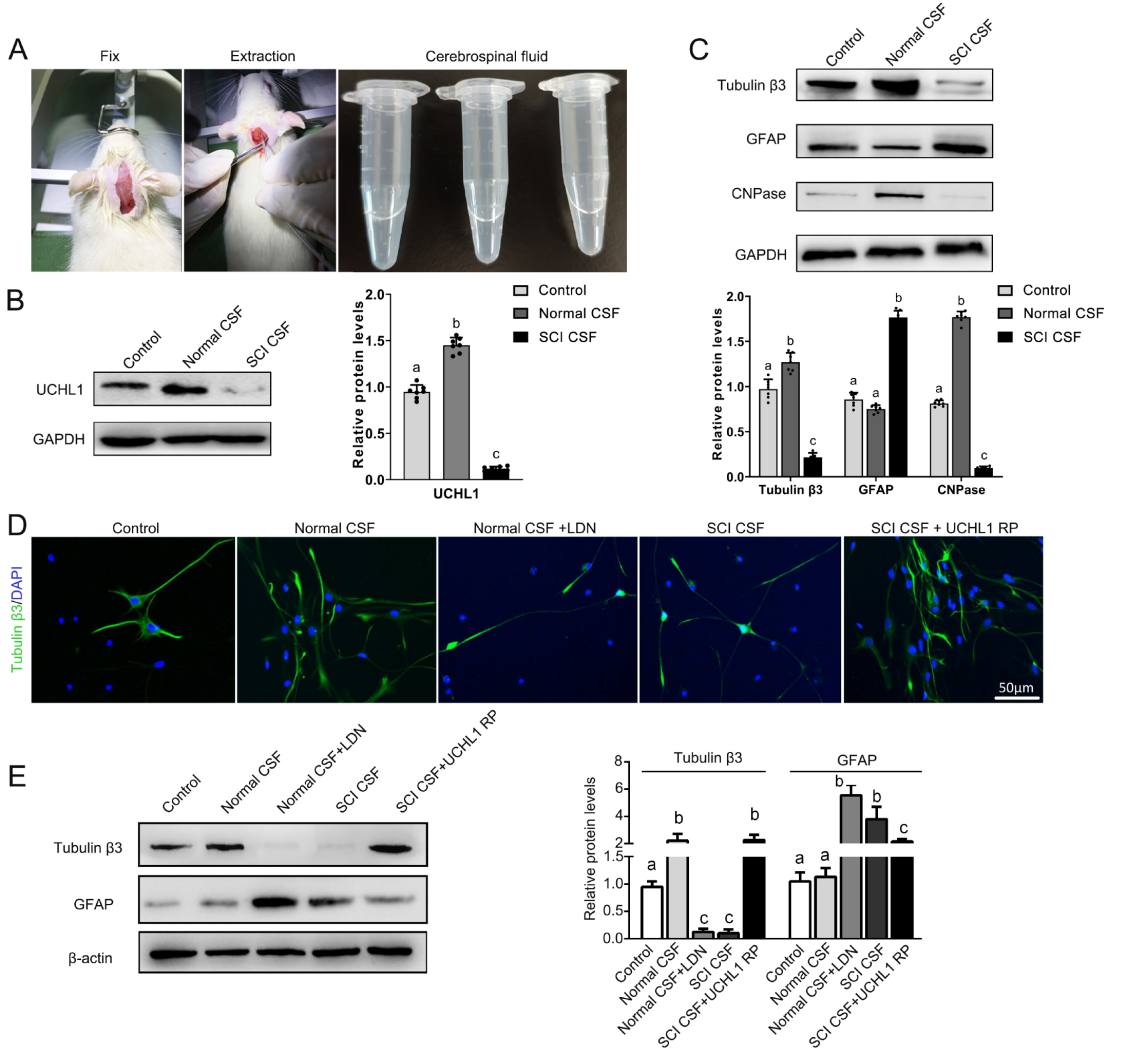
Figure 4 SCI CSF inhibits UCHL1 expression and NSC differentiation into neurons (A) CSF was collected via a cisternal puncture at the cerebellopontine cistern. Twenty-four hours post-surgery, rats from both the sham and SCI groups were immobilized in a stereotaxic apparatus, and CSF samples were obtained from the cerebellomedullary cisterna of both the control and SCI groups. (B,C) Western blot analysis revealed the expression levels of UCHL1, Tubulin-β3, GFAP, and CNPase after culture with 10% CSF for 7 days. (D,E) Immunofluorescence staining and western blot analysis were performed to assess the impact of SCI CSF on NSC differentiation. Different groups of lowercase letters (a, b, c) represent significant differences (P < 0.05). n = 7 per group. Scale bar = 50 μm.

Twenty-four hours post-SCI, CSF was collected via a cisterna magna puncture via a stereotactic apparatus ( Figure 4A). NSCs were subsequently exposed to 10% artificial CSF, normal CSF, and SCI CSF for a period of 7 days. Exposure to normal CSF resulted in increased expression of UCHL1 and neuronal-specific and oligodendrocyte-specific proteins while simultaneously decreasing the levels of astrocytic proteins. Conversely, exposure to SCI CSF produced the opposite effects ( Figure 4B,C). After 14 days of differentiation, immunofluorescence ( Figure 4D) and western blot analysis ( Figure 4E) revealed that normal CSF promoted NSC differentiation into neurons and oligodendrocytes but inhibited astrocyte differentiation. In contrast, SCI-induced CSF inhibited neuronal differentiation. These data suggest that components within SCI CSF adversely regulate UCHL1 expression, thereby hindering neuronal differentiation.

### Upregulation of miR-146a-3p in CSF exosomes after SCI

**Figure 5:**
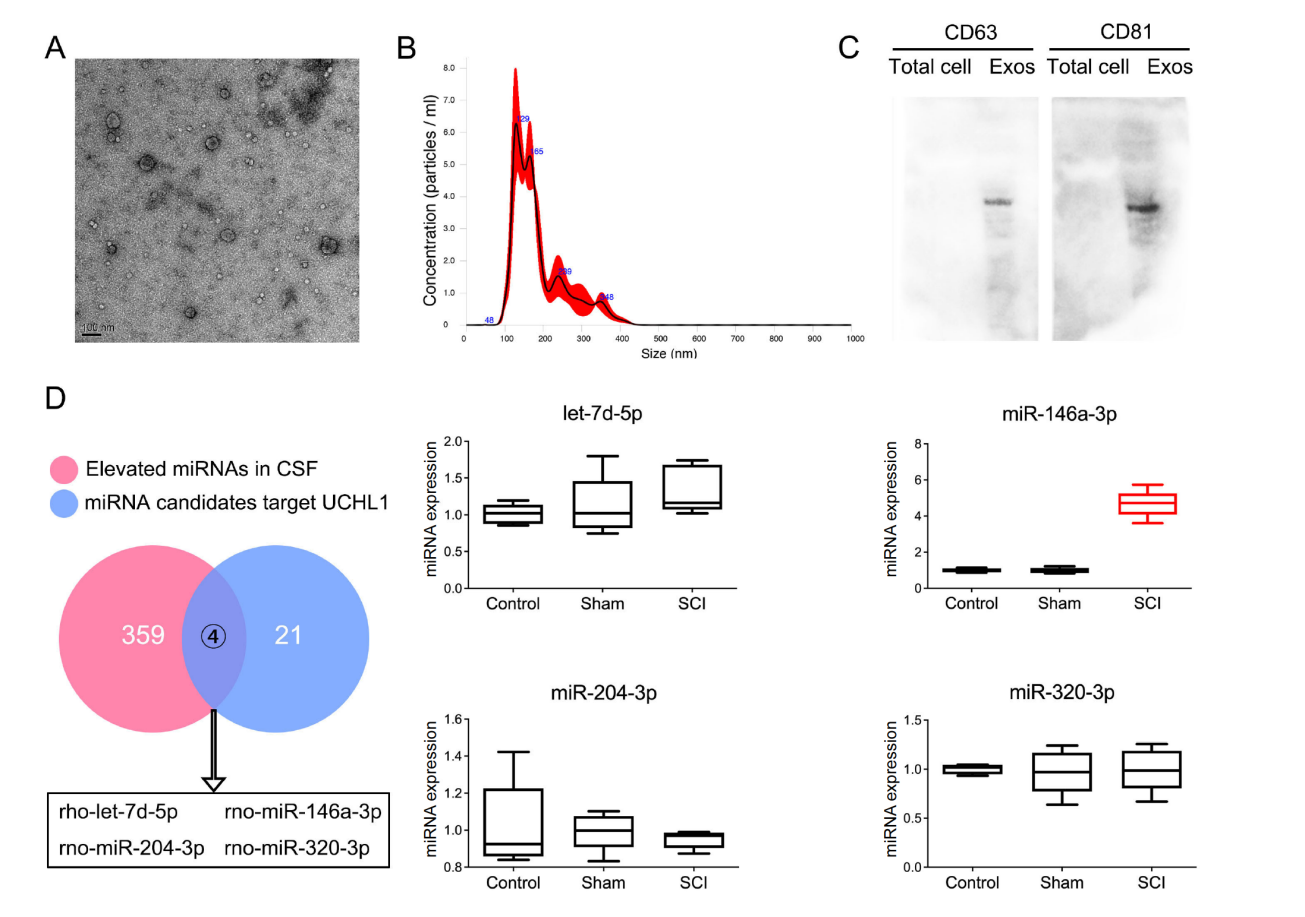
Figure 5 Identification and miRNA analysis of SCI CSF exosomes (A) Detection of exosome morphology by transmission electron microscopy. (B) The diameter of the exosomes was measured by NTA. (C) Western blot analysis was conducted to evaluate the expression levels of exosomal marker proteins in both total cell lysates and isolated exosomes. (D) miR-146a-3p was highly expressed in SCI CSF. A Venn diagram illustrating the intersection of elevated miRNAs in SCI CSF and candidate miRNAs targeting UCHL1, and 4 candidate miRNAs in exosomes were quantified using RT-qPCR.

Twenty-four hours after the surge, CSF exosomes were isolated from both normal and SCI rats and characterized. Transmission electron microscopy revealed that the extracted exosomes exhibited a characteristic cup-shaped vesicle morphology ( Figure 5A). Nanoparticle tracking analysis revealed that the diameter of the extracted exosomes ranged primarily from 120 to 160 nm ( Figure 5B). The exosome marker proteins CD63 and CD81 were also highly expressed ( Figure 5C).

The miRNA expression data for the CSF of SCI patients were obtained from the GEO database (GSE19890), which revealed that 359 miRNAs were significantly upregulated postinjury. Using the miRWalk database, we predicted miRNAs capable of targeting the rat *UCHL1* 3′UTR, identifying 21 candidates with binding *P* values exceeding 0.9. Venn diagram analysis revealed an intersection of the 4 miRNAs between these two datasets ( Figure 5D). We quantified the expression levels of these 4 miRNAs in exosomes using RT-qPCR. The results demonstrated that miR-146a-3p was significantly upregulated in exosomes isolated from SCI CSF compared with those isolated from both the healthy control and sham operation groups ( Figure 5D). These findings suggest that the elevated expression of miR-146a-3p in the CSF after SCI may decrease the level of UCHL1 and impair neuronal regeneration.

### miR-146a-3p targets and inhibits *UCHL1* mRNA

**Figure 6:**
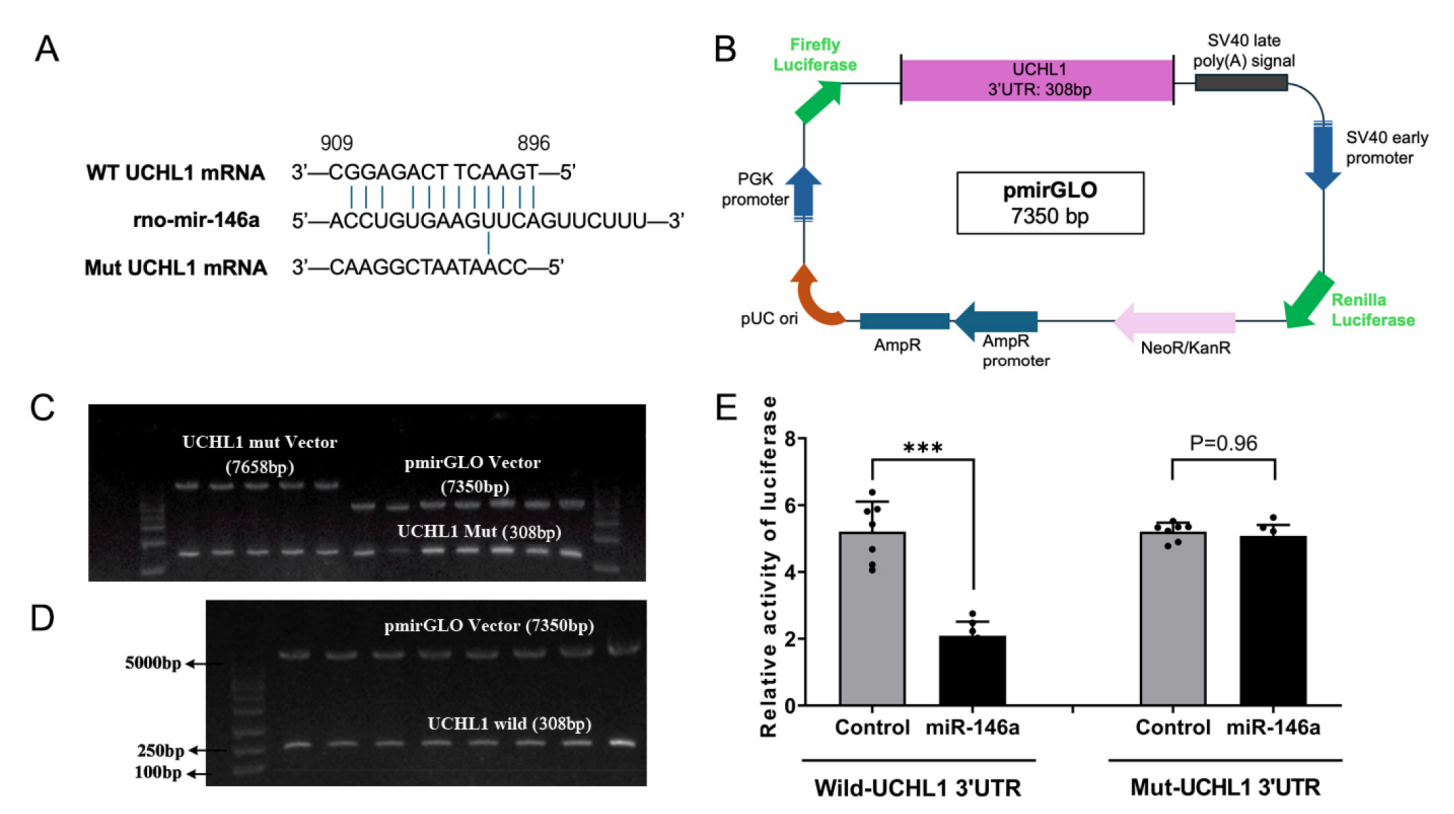
Figure 6 Construction of the UCHL1 dual-luciferase reporter system and miR-146a-3p targeting analysis (A) miR-146-3p binding site and mutation sequence in the UCHL1 3′UTR. (B) Structural diagram of the double-fluorescence reporter plasmid. (C) Agarose gel electrophoresis analysis of the dual-fluorescence reporter plasmid. Left panel: direct electrophoretic image following plasmid connection. Right panel: electrophoretic image of the connected plasmid of Mut UCHL1 after double enzyme digestion. (D) Connected WT UCHL1 plasmid after double enzyme digestion. (E) Effect of miR-146a-3p on the fluorescence activity of the WT and Mut UCHL1 3′UTRs.

To validate the interaction between miR-146a-3p and UCHL1, a dual-luciferase reporter assay incorporating the WT or Mut 3′UTR of *UCHL1* mRNA was used ( Figure 6A). The fluorescence reporter system consists of a 7350 bp pmirGLO plasmid and an inserted 308 bp WT or Mut 3′UTR of the *UCHL1* gene ( Figure 6B). Following the integration of the *UCHL1* sequence into the reporter vector, agarose gel electrophoresis was conducted. The results confirmed the successful construction of the dual fluorescence reporting system for the *UCHL1* sequence (left side, Figure 6C; 7658 bp). Subsequent double enzyme digestion of the recombinant plasmids yielded target fragments of 308 bp and 7350 bp (right side, Figure 6C,D). Sequencing following double enzyme digestion confirmed that the obtained fragment was indeed the desired target. Ultimately, dual-fluorescence reporter assays were conducted on HEK293T cells. The results demonstrated that miR-146-3p significantly decreased the fluorescence activity of the WT *UCHL1* 3′UTR by 58.7%, whereas it had no significant effect on the Mut *UCHL1* 3′UTR ( Figure 6E). These findings confirmed that miR-146-3p directly interacts with the 3′UTR of *UCHL1* mRNA, resulting in its downregulation.

### miR-146a-3p inhibits neuron regeneration

**Figure 7:**
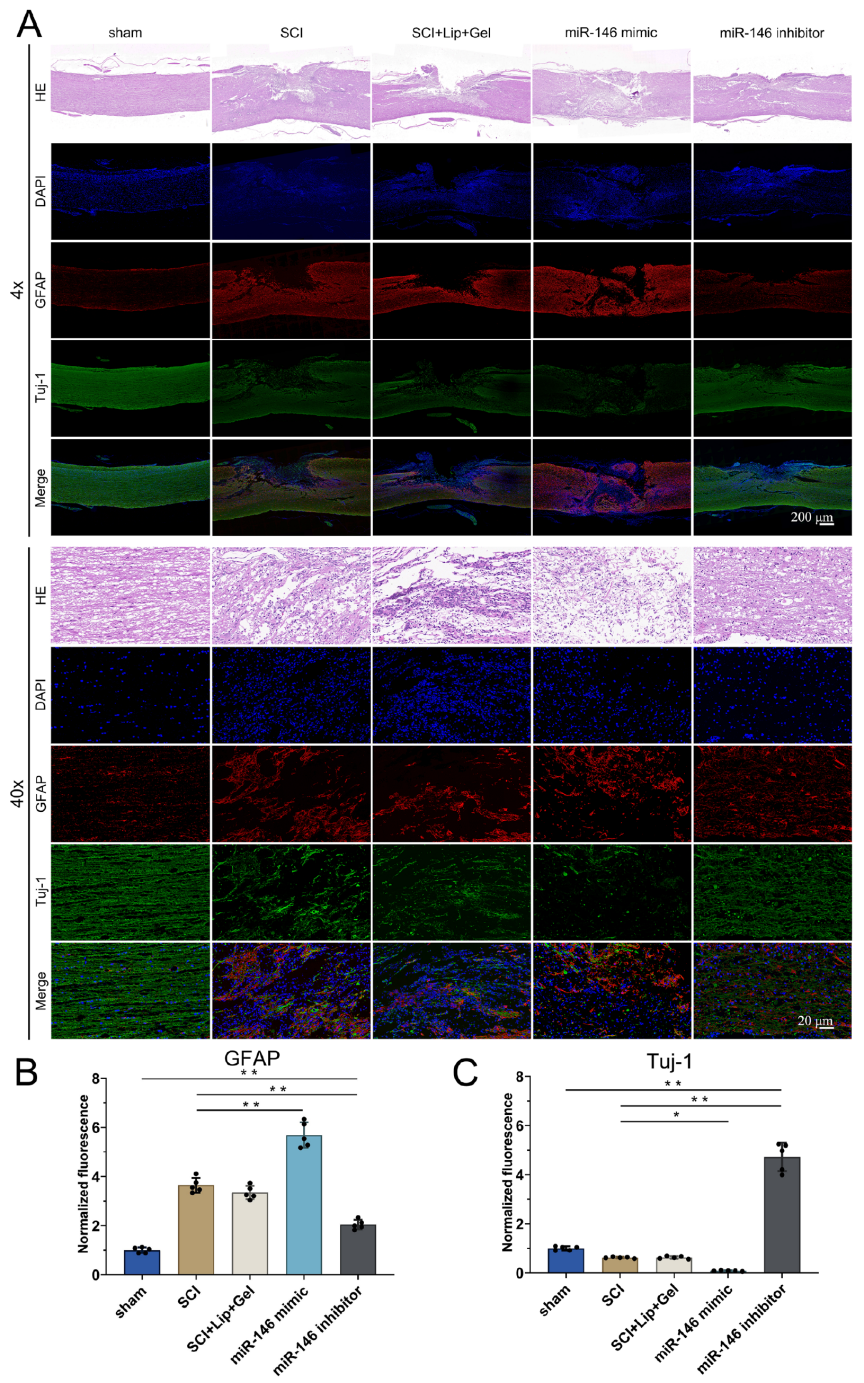
Figure 7 miR-146a-3p inhibits neuron regeneration The SCI rats were divided into the sham, SCI, SCI + Lip + Gel (SCI + Lipo2000 + Matrigel), miR-146 mimic (SCI + miR146a-3p mimic + Lipo2000 + Matrigel), and miR-146 inhibitor (SCI + miR146a-3p inhibitor + Lipo2000 + Matrigel) groups. (A) Histological images (H&E staining) of longitudinal sections of the injured spinal cord and immunofluorescence images at different magnifications on day 21 in different groups. 4×: scale bar = 200 μm; 40×: scale bar = 20 μm. (B,C) Changes in GFAP and Tuj-1 in the injured spinal cords of the other groups were measured, and the fluorescence intensity of the sham group was used as the standard. *P < 0.05, ** P < 0.01. ns: not significant. n = 5 per group.

Histological analysis revealed the significant influence of miR-146a-3p on neuronal regeneration following SCI. H&E staining of longitudinal spinal cord sections at 21 days post-injury revealed substantial enlargement of the cavity area at the injury site in the miR-146a mimic group. This increase was associated with a notable increase in the number of GFAP-positive astrocytes, indicative of enhanced glial scar formation, which impedes the regenerative processes. In contrast, the miR-146a-3p inhibitor group exhibited a marked reduction in the intermuscular space and a significant improvement in Tuj-1-positive neurons, underscoring the pivotal role of miR-146a-3p in hindering neuronal differentiation and regeneration. Immunofluorescence assays further confirmed these observations, demonstrating the adverse effects of miR-146a-3p on neuronal differentiation. Tuj-1, a specific neuronal marker, showed reduced expression in the miR-146a mimic group, whereas an increase in GFAP expression highlighted the promotion of astrocyte differentiation over neuronal lineage commitment ( Figure 7A–C). In contrast, the group treated with the miR-146a-3p inhibitor demonstrated enhanced neuronal differentiation, as indicated by an increased number of Tuj-1-positive cells. These results support the hypothesis that miR-146a-3p, through the modulation of UCHL1 expression, plays a direct role in influencing the equilibrium between neuronal and astrocytic differentiation in SCI. The inhibition of miR-146a-3p effectively reduces glial scar formation and promotes neuronal regeneration, thereby providing strong evidence for its potential as a therapeutic target in SCI recovery.

### miR-146a-3p impairs motor function recovery

**Figure 8:**
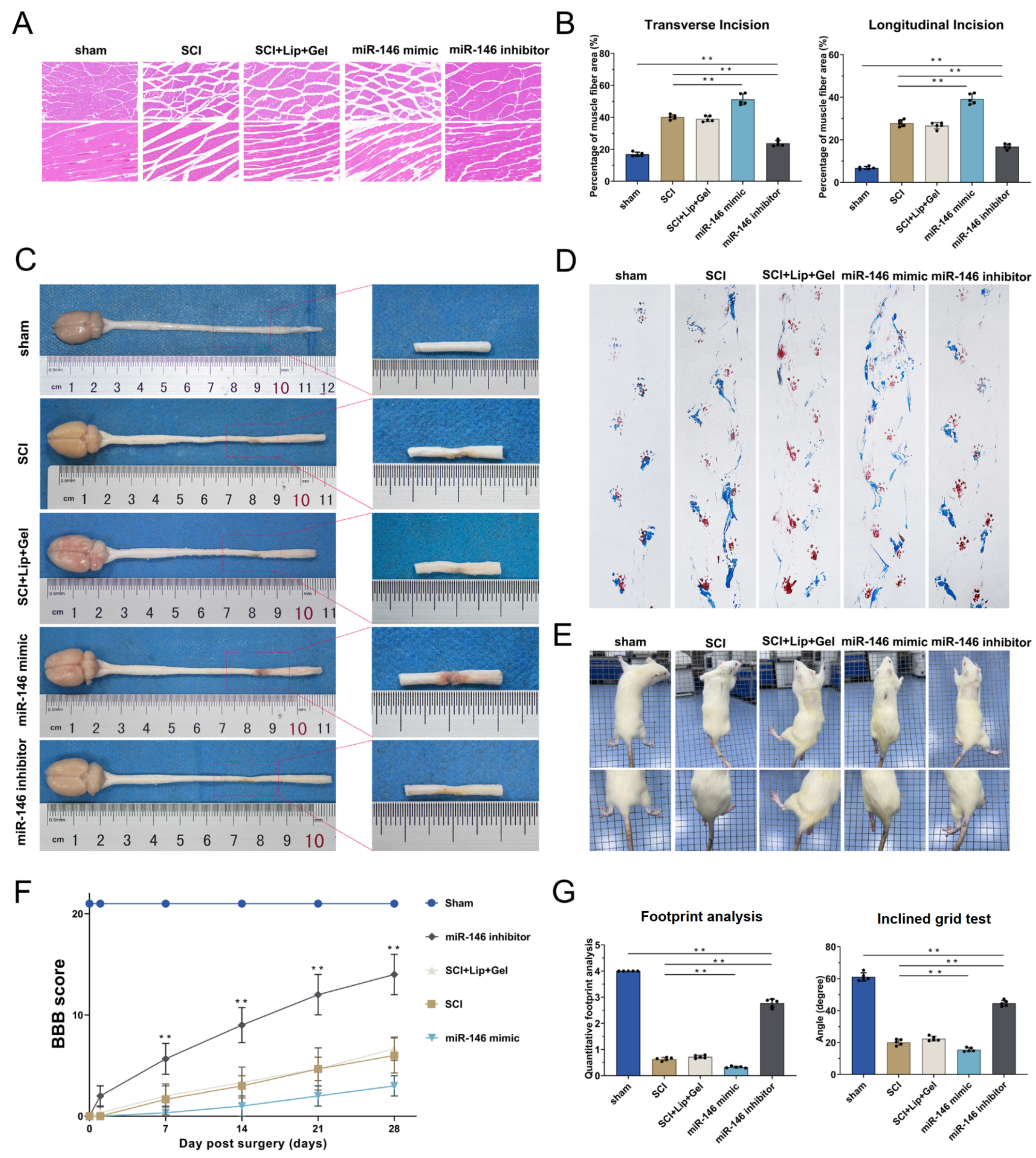
Figure 8 miR-146a-3p impairs motor function recovery The SCI rats were divided into the sham, SCI, SCI + Lip + Gel (SCI + Lipo2000 + Matrigel), miR-146 mimic (SCI + miR146a-3p mimic + Lipo2000 + Matrigel), and miR-146 inhibitor (SCI + miR146a-3p inhibitor + Lipo2000 + Matrigel) groups. (A) H&E staining of both transverse and longitudinal sections of the gastrocnemius muscles at 21 days. (B) Statistical chart of the intermuscular space at the transverse and longitudinal incisions. (C) Spinal cord morphology was photographed. (D–G) Footprint analysis (D,G), inclined grid test (E), and BBB scoring (F) were conducted to measure the recovery of motor ability in the rats. *P < 0.05, **P < 0.01. ns: not significant. n = 5 per group.

At 21 days post-surgery, H&E staining of both transverse and longitudinal sections of the gastrocnemius muscles in rat hind limbs demonstrated that miR-146a-3p led to an increased intermuscular space, which is indicative of more pronounced muscle atrophy. Conversely, the administration of a miR-146a inhibitor resulted in a significant reduction in the intermuscular space and facilitated the recovery of hind limb function ( Figure 8A,B). Additionally, the results of the morphological examination of the spinal cord indicated that miR-146a-3p interfered with the healing process following SCI ( Figure 8C). Footprint analysis revealed that footprint quantity and the foot angle of the hind limb in the rats treated with miR-146a-3p were significantly lower than those in the controls, whereas hind limb function in the miR-146a inhibitor group showed signs of recovery ( Figure 8D,E,G). These findings were corroborated by the BBB scores ( Figure 8F).

## Discussion

SCI represents a critical condition of the CNS, frequently leading to enduring neurological impairments that substantially diminish patients’ quality of life and life expectancy. In recent decades, substantial advancements have been achieved in SCI research, with efforts concentrated on elucidating the mechanisms of injury, devising neuroprotective strategies, investigating regenerative repair techniques, and enhancing functional recovery [ 25, 26]. SCI encompasses not only primary mechanical trauma but also intricate secondary injury processes, including inflammatory responses, excitotoxicity, free radical generation, and apoptosis. Secondary processes exacerbate damage to neurons and glial cells, thereby hindering neural regeneration.

To address secondary injury, a range of neuroprotective agents have been explored. Methylprednisolone, a glucocorticoid, has been extensively utilized in the treatment of acute SCI to attenuate inflammation and oxidative stress [27]. Although methylprednisolone has demonstrated some efficacy in the treatment of acute SCI, its efficacy remains a subject of debate, as it may be linked to considerable side effects [28]. To address the limitations associated with methylprednisolone treatment, researchers have investigated alternative therapeutic approaches. For example, ezetimibe (EZE) has demonstrated potential in reducing inflammation and oxidative stress following spinal cord injury by modulating the AMPK/Nrf2 pathway, thereby facilitating functional recovery [29]. Furthermore, certain antioxidants and anti-inflammatory drugs have demonstrated potential protective effects. Stem cell therapy is considered a promising strategy for enhancing neural regeneration and functional recovery. In particular, NSCs, mesenchymal stem cells, and induced pluripotent stem cells (iPSCs) have been demonstrated to enhance axonal regeneration, secrete neurotrophic factors, and modulate immune responses [30]. Biocompatible materials, such as collagen, gelatin, and poly(lactic-co-glycolic acid), have been employed to construct neural scaffolds that facilitate and direct the growth of new axons. These scaffolds can be integrated with cells and growth factors to create tissue-engineered grafts, thereby promoting spinal cord regeneration [31]. Additionally, gene therapy and molecular regulation strategies, including gene editing technologies such as CRISPR-Cas9, have been utilized to modulate the expression of key genes in SCI [32]. For example, the suppression of the expression of the myelin-associated inhibitor Nogo-A has been shown to facilitate axonal regeneration [33]. Nonetheless, despite the significant potential of CRISPR-Cas9 technology, several challenges persist, including the need to increase editing efficiency and minimize off-target effects to ensure its safety and efficacy in clinical applications. Similarly, the modulation of microRNA expression is regarded as a critical approach for influencing neural regeneration [34] .

Regulating the microenvironment following SCI is essential for facilitating neural regeneration, given that injury precipitates complex alterations that impede repair processes. In the aftermath of injury, the local environment undergoes activation of inflammatory responses, characterized by the infiltration of neutrophils, macrophages, and activated microglia, which release proinflammatory cytokines, including TNF-α, IL-1β, and IL-6, thereby exacerbating secondary damage [35]. Furthermore, glial scar formation ensues as a result of the proliferation of activated astrocytes and fibroblasts, which produce extracellular matrix proteins such as chondroitin sulfate proteoglycans (CSPGs), creating physical and chemical impediments to axonal growth [ 36, 37]. Inhibitory molecules such as Nogo-A, MAG, and OMgp are overexpressed, which directly hinders axonal regeneration [38]. Additionally, there is a deficiency of supportive elements, including neurotrophic factors such as BDNF and NT-3 [39]. The regulation of the microenvironment, which involves the inhibition of glial scar formation, the modulation of inflammatory responses, and the promotion of angiogenesis, is crucial for neural regeneration. To address these challenges, strategies have been developed to modulate the microenvironment by suppressing detrimental elements, such as inflammatory cytokines and inhibitory molecules, while simultaneously introducing supportive components such as neurotrophic factors and biocompatible scaffolds [40]. For instance, early surgical decompression and stringent blood pressure management are crucial strategies for enhancing neurological function [41]. Furthermore, the integration of cell therapy and growth factors is also viewed as a potential avenue for future SCI treatment [42]. By integrating these approaches, a more favorable environment for neural regeneration can be established, thereby increasing the potential for functional recovery in patients with SCI. Despite the promising outcomes demonstrated by numerous experimental treatments in animal models, the translation of these therapies into clinical applications continues to pose significant challenges [42]. Emerging treatments, including stem cell transplantation and functional electrical stimulation, are currently undergoing clinical trials, but the current clinical trial results are still inconsistent [43]. Therefore, by synthesizing diverse research findings and advancing our understanding of the mechanisms underlying SCI, more effective treatment options can be developed. These advancements are expected to lead to the development of efficacious therapeutic strategies aimed at promoting spinal cord regeneration and functional recovery.

Recent studies have demonstrated that miRNAs, such as miR-146a-3p, play crucial roles in regulating inflammatory responses following SCI. For example, a study by Sun *et al*. [44] revealed that miR-146a-3p and miR-202-3p attenuate inflammation by inhibiting key molecules such as TLR4, IRAK1, and TRAF6 in SCI rat models. This finding is consistent with our results. Our study provides a comprehensive analysis of the molecular mechanisms impeding neuronal regeneration following SCI, with a particular emphasis on the facilitation of the miR-146a/UCHL1 axis by CSF exosomes. Our findings revealed that miR-146a-3p, which is markedly upregulated in CSF exosomes post-SCI, directly inhibits the expression of UCHL1, a critical regulator of NSC differentiation into neurons. This suppression is consistent with the observed inhibition of neuronal differentiation and the concomitant promotion of astrocytic differentiation, processes that contribute to fibrotic scar formation and impede neural regeneration.

These findings largely support our hypothesis. The identification of miR-146a-3p as a principal inhibitory factor in SCI CSF aligns with our bioinformatics predictions and was validated *in vitro* using dual-luciferase reporter assays. Moreover, *in vivo* overexpression of UCHL1 markedly enhanced neuronal differentiation and functional recovery, corroborating the findings of previous studies that emphasized the role of UCHL1 in neurogenesis. These results highlight the adverse effects of the miR-146a-3p-mediated suppression of UCHL1 on the regenerative microenvironment of the spinal cord. Our findings are consistent with the literature that emphasizes the role of exosomal microRNAs in modulating CNS homeostasis. Research on CSF exosomal miRNAs has demonstrated their ability to influence NSC fate, particularly in the context of neurodegenerative disorders. Importantly, the role of UCHL1 in promoting neuronal differentiation has been well documented in other neurological conditions, such as Parkinson’s disease, where its dysregulation is implicated in pathogenesis. Our study advances the current understanding by concentrating on the microenvironment specific to SCI, emphasizing the systemic impact of CSF components beyond the immediate site of injury.

Contrary to expectations, our findings demonstrate a significantly greater inhibitory effect of SCI CSF on NSC neuronal differentiation than previously anticipated. This phenomenon may be attributed to the synergistic effects of various microRNAs or proteins present in SCI CSF, which necessitates further investigation. Furthermore, the behavioral improvements observed in the groups overexpressing UCHL1 were modest, indicating that although UCHL1 is a crucial target, comprehensive therapeutic strategies necessitate addressing multiple components within the injury microenvironment. This study has several implications for future research and clinical practice. First, targeting miR-146a-3p via antisense oligonucleotides or exosome engineering could offer a novel therapeutic pathway for promoting neural regeneration. Second, our approach transitions the focus from localized treatments to systemic interventions, thereby addressing the broader challenges presented by the SCI microenvironment. Finally, the use of CSF as a source of diagnostic and therapeutic biomarkers represents an innovative direction for personalized medicine in SCI treatment. The BBB scoring experiment was concluded at 28 days because of logistical constraints. Although the BBB score had not yet reached a plateau, this time point was selected to balance the needs of data collection with considerations for animal welfare. *In vitro* studies demonstrated that UCHL1 overexpression enhanced the differentiation of NSCs derived from the subventricular zone (SVZ). While NSCs derived from the spinal cord may exhibit distinct differentiation patterns, the SVZ-derived model was selected because of its well-established neurogenic potential and the ease of obtaining NSCs, as the SVZ contains a rich population of readily accessible NSCs. In contrast, NSCs derived from the adult spinal cord are limited in number, are challenging to extract, and exhibit a reduced regenerative capacity. This model has been extensively utilized in studies of neurogenesis and spinal cord injury due to its practical advantages and efficacy in promoting neural repair, thus strongly supporting our selection of SVZ-derived neural stem cells as a model system [ 45– 47].

The pivotal role of miR-146a as a regulatory factor in neuropathological and injury-related processes is imperative. miR-146a is implicated in various critical functions, including synaptic dysfunction, mitochondrial abnormalities, neuronal death, and the accumulation of amyloid-beta (Aβ). Elevated levels of miR-146a have been demonstrated to downregulate the expression of essential synaptic proteins such as Synaptotagmin 1 (Syt1) and Neuroligin 1 (Nlg1), thereby disrupting synaptic function and precipitating synapse loss—a hallmark of Alzheimer’s disease (AD) [48]. This synaptic loss contributes to early cognitive decline, preceding neuronal death. Furthermore, miR-146a targets multiple mRNAs encoding proteins of the mitochondrial electron transport chain, thereby adversely affecting mitochondrial integrity and diminishing cerebral glucose metabolism, which is crucial for neuronal health. In AD models, this mitochondrial dysfunction exacerbates the disease by inducing energy deficits in neurons [49]. Additionally, miR-146a is associated with various forms of neuronal death, particularly apoptosis. It induces apoptosis by activating pathways such as signal transducer and activator of transcription 1 (STAT1) and c-Myc, both of which play critical roles in the regulation of cell death [50]. Research indicates that the overexpression of miR-146a augments neuronal apoptosis in AD models, highlighting its importance in the pathogenesis of this disease. Research indicates that overexpression of miR-146a exacerbates neuronal apoptosis in AD models, highlighting its detrimental effect on neuronal survival [51]. Additionally, miR-146a contributes to Aβ accumulation, a hallmark of AD pathology. By modulating pathways such as p-p38 and NF-κB, miR-146a facilitates Aβ deposition and reactive oxygen species (ROS) accumulation, thereby exacerbating neuroinflammation and neuronal damage [52].

In addition to miR-146a-3p, various studies have identified microRNAs that regulate UCHL1 levels. Notably, miR-922 targets the 3′UTR of *UCHL1* mRNA, inhibiting its translation and reducing UCHL1 protein levels. Overexpression of miR-922 not only diminishes UCHL1 levels but also elevates phosphorylated tau protein levels, which fosters neurofibrillary tangle formation and accelerates neuronal damage. Conversely, restoring UCHL1 expression by inhibiting miR-922 ameliorates synaptic function and mitigates cognitive impairment [53]. In an ischemic stroke model, miR-181b was found to directly target UCHL1, and the downregulation of miR-181b confers neuroprotection against ischemic injury [54]. Furthermore, another study indicated that miR-214-3p also targets and downregulates UCHL1. A separate study revealed that miR-214-3p targets and downregulates the expression of UCHL1 [55]. However, our analysis of miRNA expression data from the CSF of SCI rats (GSE19890) revealed that several previously reported miRNAs were not upregulated. Other miRNAs may also regulate UCHL1 levels in the CSF. Within the CNS, miR-146a-3p is expressed across various cell types. In microglia, miR-146a plays a role in regulating neuroinflammation and immune responses, particularly in AD models [56]. In astrocytes, miR-146a modulates inflammatory responses [57]. It is also present in neurons, where it regulates inflammatory pathways and promotes neuroprotection [58]. Furthermore, miR-146a-3p contributes to the differentiation of oligodendrocyte precursor cells, thereby supporting myelination and nerve repair [59].

This study highlights the role of the miR-146a-3p/UCHL1 axis in SCI-induced neural regeneration but has limitations. Despite the use of SVZ-derived NSCs in this study, it is crucial to recognize that spinal cord-derived NSCs may respond differently to SCI in terms of their differentiation potential. Future research should incorporate spinal cord-specific NSCs to more accurately reflect neurogenesis induced by injury. Additionally, the reliance on Sprague-Dawley rats restricts their generalizability to humans because of differences in immune response and CSF composition. The focus on miR-146a-3p overlooks potential synergistic effects of other exosomal miRNAs and proteins in CSF, leaving a broader molecular network unexplored. Behavioral improvements following UCHL1 overexpression were modest, suggesting that additional factors in the SCI microenvironment influence neuronal regeneration. Moreover, exosome isolation methods lack specificity, potentially obscuring the roles of cell type-specific exosomes and miR-146a-3p. Future studies should incorporate human-derived models, explore other molecular pathways, and refine exosome analysis to increase the understanding and translational potential of miR-146a-3p UCHL1-targeted therapies for SCI. In addition, *in vivo* validation of the miR-146a-3p/UCLH1 axis would increase the robustness of our findings and strengthen our conclusions. Moreover, we acknowledge that CSF contains not only miRNAs but also numerous small peptides, proteins, metabolites, and other potential substances that may influence UCHL1 levels. A significant limitation of this study is its exclusive focus on miRNA changes, which precluded the exploration of these alternative regulatory mechanisms. Additionally, since CSF is produced primarily in the choroid plexus within the ventricles and circulates throughout the CNS, alterations in neurons, microglia, and astrocytes within the brain or spinal cord microenvironment may impact CSF composition. Thus, identifying the precise anatomical origin and cellular source of miR-146a-3p is a complex and extensive research challenge that we plan to address in future studies.

This study elucidates the pivotal role of the miR-146a-3p/UCHL1 axis in modulating NSC differentiation and its subsequent effects on neural regeneration following SCI. Our findings reveal that exosomes derived from CSF in SCI significantly increase miR-146a-3p levels, which in turn directly repress the expression of UCHL1, a crucial promoter of neuronal differentiation. This regulatory mechanism impedes the differentiation of NSCs into neurons, promotes astrocytic lineage commitment, and facilitates scar formation, thereby obstructing neural regeneration. Through the overexpression of UCHL1, we demonstrated a marked increase in the neuronal differentiation of NSCs and functional recovery, confirming its therapeutic potential. These results address this research question by elucidating the mechanism through which miR-146a-3p in SCI CSF impairs NSC fate via the downregulation of UCHL1, thus providing insights into the molecular impediments to neural regeneration. This study lays the groundwork for the development of novel therapeutic strategies targeting miR-146a-3p and UCHL1 to improve recovery outcomes following SCI.
